# Genetic alterations in myeloproliferative and myelodysplastic/myeloproliferative neoplasms – a practical guide to WHO-HAEM5

**DOI:** 10.1515/medgen-2024-2003

**Published:** 2024-03-06

**Authors:** Constanze Kühn, Katharina Hörst, Hans M. Kvasnicka, Andreas Hochhaus, Andreas Reiter

**Affiliations:** Universitätsklinikum Jena Abteilung Hämatologie und Internistische Onkologie, Klinik für Innere Medizin II Jena Germany; MLL – Munich Leukemia Laboratory Munich Germany; MLL – Munich Leukemia Laboratory Munich Germany; University Hospital Institute for Pathology and Molecular Pathology Wuppertal Germany; III. Medizinische Klinik Medical Clinic for Haematology and Oncology, University Medical Centre Mannheim Mannheim Germany

**Keywords:** WHO, classification, HAEM5, myeloproliferative neoplasms, myelodysplastic /myeloproliferative neoplasms, overlap

## Abstract

Within the World Health Organization (WHO) classification of haematopoietic neoplasms, particularly its fifth version from 2022 (WHO-HAEM5), myeloid neoplasms are not only grouped into myeloproliferative (MPN) and myelodysplastic neoplasms (MDS). There is also a group of haematological disorders that share features of both categories termed myelodysplastic /myeloproliferative neoplasms (MDS/MPN). In this article, we aim to provide a comprehensive and practical guide to WHO-HAEM5 highlighting the genetic alterations that underlie MPN and MDS/MPN. This guide provides an overview of the overlapping commonalities among these entities, as well as their unique characteristics.

## Introduction

Myeloproliferative neoplasms (MPN) and myelodysplastic/myeloproliferative neoplasms (MDS/MPN) represent a heterogeneous group of haematological disorders characterized by clonal expansion and dysregulation of myeloid cell proliferation. These conditions have been the focus of extensive research due to their complex genetic landscapes, clinical diversity, and diagnostic challenges.

Within the spectrum of myeloid neoplasms, distinguishing between myelodysplastic neoplasms (MDS) with variable cytopenias and MPN with variable cytosis is a pivotal diagnostic and prognostic consideration. MDS is characterized by ineffective haematopoiesis, leading to peripheral cytopenia, while MPN is mainly characterized by the excessive production of mature myeloid cells, resulting in peripheral cytosis. Understanding the genetic basis of MDS and MPN is crucial for improving diagnosis, predicting prognosis, and developing customized therapeutic strategies [1].

## Myeloproliferative neoplasms (MPN)

The abnormal proliferation of one or more terminally differentiated myeloid cell lines in the peripheral blood define the heterogeneous group of MPN [1]. They were first classified based on the presence or absence of the *BCR*::*ABL1* fusion gene that originates from the Philadelphia (Ph) chromosome (aberrant chromosome 22) which typically results from the reciprocal translocation between chromosome 9 and chromosome 22 [t(9;22)(q34.1;q11.2)]. Chronic myeloid leukaemia (CML) represents the only *BCR*::*ABL1*-positive MPN [1, 2].

### *BCR*::*ABL1*-positive MPN: chronic myeloid leukaemia (CML)

A characteristic t(9;22)(q34.1;q11.2) is present in 90–95 % of CML patients, while the remaining cases have either variant translocations that involve a third or even a fourth chromosome in addition to chromosomes 9 and 22, or a cytogenetically cryptic rearrangement involving 9q34.1 and 22q11.2 that cannot be identified by chromosome banding analysis (CBA). In such cases, the *BCR*::*ABL1* fusion gene can be detected by Fluorescence in situ hybridization (FISH) analysis and/or reverse-transcriptase PCR (RT-PCR) [1]. The *BCR*::*ABL1* gene codes for fusion proteins with an activated tyrosine kinase activity [2]. CML is defined by the following criteria according to WHO-HAEM5 [1]:

Essential:

Peripheral blood leucocytosis
Detection of Philadelphia (Ph) chromosome and/or *BCR*::*ABL1* by cytogenetic and/or appropriate molecular genetic techniques

Desirable:

Bone marrow aspiration to confirm disease phase, bone marrow biopsy if findings in the peripheral blood are atypical or if a cellular aspirate cannot be obtained

The untreated disease follows a biphasic course: CML manifests with an initial indolent chronic phase (CP) with neoplastic cells mostly confined to the blood, bone marrow, spleen and liver. This is followed by the blast phase (BP) in bone marrow and/or any extramedullary site. The previously called accelerated phase (AP) has been omitted in WHO-HAEM5 because it is less useful in the tyrosine kinase inhibitor (TKI) era [1]. CML with specific risk factors is now called ‘high-risk chronic phase’ [1].

### *BCR*::*ABL1* detection is essential to the diagnosis of CML

#### CML: cytomophology

Cytomorphology is important in the diagnosis of CML and contributes significantly to the differentiation of CML from other myeloproliferative disorders. At diagnosis of CML a bone marrow aspirate is required for cytomorphology to distinguish CP from BP depending on blast percentage [3].

#### CML: cytogenetics

Since CML is defined by the presence of either the Philadelphia chromosome or the *BCR::ABL1* fusion gene, its detection is the first priority in the diagnostic workflow. Cytogenetics should be performed by CBA from bone marrow cells [3]. FISH can be used for confirming presence or absence of a rearrangement of *BCR* or *ABL1* or a* BCR*::*ABL1* fusion gene. In absence of a Philadelphia chromosome, FISH analysis is recommended according to the European LeukaemiaNet (ELN) recommendation [3].

The detection of additional chromosomal aberrations besides t(9;22) at diagnosis or during the disease course can have a major impact on prognosis. Therefore, ELN distinguishes between “high-risk” and “low-risk” additional aberrations. “High-risk” additional aberrations include trisomy 8 (in combination with others), trisomy 19, a second Philadelphia chromosome, i(17q), –7/del(7q), aberrations of 11q23 and 3q26.2, and a complex karyotype. All other additional aberrations are assigned to the “low-risk” group. Testing for additional aberrations is indicated at initial diagnosis, treatment failure/resistance, and (suspected) progression [3].

#### CML: molecular genetics

Molecular genetics, using PCR and sequencing methods, plays an important role in the diagnosis of CML. *BCR*::*ABL1* exists in several different isoforms depending on the precise position of genomic breakpoints at 9q34.1 and 22q11.2 [1]. A qualitative reverse RT-PCR is mandatory for the identification of the type of *BCR*::*ABL1* transcripts and adequate response assessment on TKI therapy [3]. Of note, about 2–4 % of patients harbour atypical *BCR*::*ABL1* transcripts that may lead to false negative results using routine primer/probes [3]. In addition to the detection of the *BCR*::*ABL1* fusion gene and determination of transcript type, molecular genetics are routinely used both for response monitoring and identification of mutations conferring resistance in case of treatment failure.

Quantifying treatment response in CML is performed by real-time PCR. Therefore, *BCR*::*ABL1* expression is quantified relative to a reference gene (usually *ABL1* or *GUSB*) and standardized according to the International Scale (IS). By this, the assessment of molecular response (MR) and the establishment of defined milestones such as the major molecular response (MMR) are enabled [3].

#### CML: therapy & prognosis

Due to the use of TKIs in recent years, the life expectancy of patients with CML is almost comparable to a healthy control population [4]. The molecular response criteria are crucial for therapy planning, which today also includes the possibility of therapy-free remission. Sensitive molecular genetic monitoring is essential, both to assess eligibility for TKI discontinuation and to detect molecular relapses at an early stage [5].

During treatment with TKIs, there is a risk of TKI resistance. The emergence of subclones of leukemic progenitor cells with *BCR*::*ABL1* point mutations may lead to altered amino acids, thereby preventing the binding of the inhibitor. Second and third generation TKIs can circumvent this type of drug failure. Furthermore, a new class of STAMP (Specifically Targeting the ABL Myristoyl Pocket) inhibitors was introduced for CML-therapy that have different binding capacity and mode of action compared to the ATP-competitive second and third generation TKIs [1]. Various scores for the risk assessment in CML have been developed. The ELN recommends using the EUTOS long-term survival (ELTS) score for risk stratification, as it holds the highest prognostic relevance. [3].

### *BCR*::*ABL1*-negative MPN

**Figure 1:**
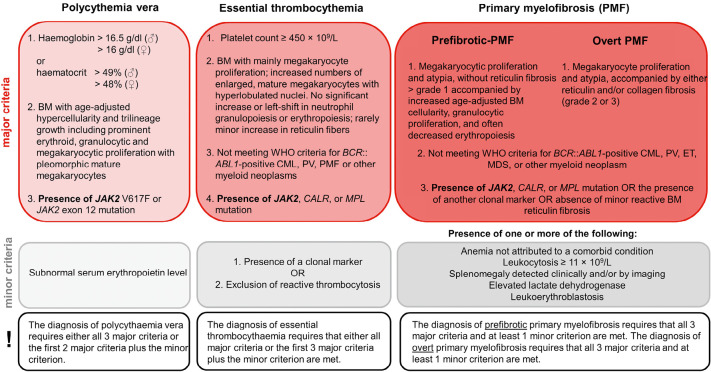
Diagnostic criteria for polycythaemia vera (PV), essential thrombocythemia (ET), and primary myelofibrosis (PMF) according to WHO-HAEM5 [1].

While the *BCR*::*ABL1* fusion gene is paramount in CML*,* this is not the case for *BCR*::*ABL1* negative MPN which encompass a group of clonal haematopoietic stem cell disorders that share some similarities besides having distinct clinical presentations. This chapter focuses primarily on the classical *BCR*::*ABL1*-negative MPN, namely polycythaemia vera (PV), essential thrombocythemia (ET), and primary myelofibrosis (PMF). Central to the understanding of these disorders is the recognition of activated *JAK2*-signaling as a common molecular hallmark [1]. The diagnostic criteria of those three MPN are summarized in Fig. 1.

### *BCR*::*ABL1*-negative MPN: cytomorphology & histopathology

In the diagnostic work-up of *BCR*::*ABL1*-negative MPN, cytomorphology and particularly histopathology of the bone marrow play a pivotal role. Morphological assessment includes evaluation of cellularity, qualitative and quantitative changes of individual cell lines, grading of fibrosis and assessment of blast counts. These parameters help to distinguish between different subtypes of MPN and to differentiate them from reactive haematopoietic changes. [1]. Accordingly, bone marrow fibrosis grade ≥2 serves as required diagnostic criteria for post-PV MF and post-ET MF [6]. Although bone marrow assessment is required for diagnosis, the quality and expertise regarding assessment of bone marrow is extremely heterogeneous. The assessment harbours limitations that have to be considered in clinical practice. The absence of crucial elements, including fibrosis grade, blast percentage, and hyperproliferation of specific lineages, within bone marrow biopsy reports can impede accurate diagnosis. Additionally, potential discrepancies may emerge as histology reports sometimes denote diagnostic subtypes inconsistent with the observed clinical findings.

### *BCR*::*ABL1*-negative MPN: cytogenetics

Chromosomal alterations in *BCR*::*ABL1*-negative MPN exhibit considerable heterogeneity, with their prevalence varying among the different MPN subtypes. These alterations are most frequently observed in PMF, followed by PV, while they are rarely found in ET. The diverse spectrum of chromosomal abnormalities reflects the clonal evolution and genomic instability inherent to these disorders. The occurring alterations include, but are not limited to, trisomy 8, del(20q), trisomy 9 and del(5q) [1, 7–11]. Different risk stratification scores exist for PMF, including the International Prognostic Scoring System (IPSS) and several updates, such as the DIPSS plus [12–19]. Within these scores, cytogenetic aberrations are differentiated according to their risk profile. Aberrations with a very high risk include for example single or multiple abnormalities of monosomy 7, inv(3)/3q21, i(17q), 12p-/12p11.2, 11q-/11q23, and autosomal trisomies other than +8 or +9 (e. g. +21, +19) [11].

The presence or absence of certain chromosomal changes can have diagnostic and prognostic implications, making their assessment a valuable part of the overall evaluation of MPN patients [7].

### *BCR*::*ABL1*-negative MPN: molecular genetics

Molecular diagnostics have emerged as the cornerstone of MPN diagnosis, with their ability to not only exclude a *BCR*::*ABL1* fusion gene but also to differentiate MPN from reactive haematological changes and to determine disease progression. Key driver genes in *BCR*::*ABL1*-negative MPN include *JAK2*, *CALR*, and *MPL*, each one contributing to the dysregulated *JAK2*-signaling pathway [1]. Next-generation sequencing (NGS) has become one of the most important tools in the molecular diagnosis of MPN, enabling comprehensive profiling of somatic mutations and aiding in risk stratification and therapeutic decision making (Table 1).

### Other *BCR*::*ABL1*-negative MPN

While PV, ET, and PMF represent the classical *BCR*::*ABL1*-negative MPN, there are several other distinct entities within this category. Each of these conditions has distinct genetic and clinical characteristics that highlight the heterogeneity of MPN.

**Chronic Neutrophilic Leukaemia (CNL):** CNL is a rare myeloproliferative neoplasm characterized by sustained neutrophilic leukocytosis in the peripheral blood. The hallmark of CNL is an elevated absolute neutrophil count (ANC) of greater than 25 × 10^9^/L. CNL patients typically lack the *JAK2*, *CALR*, or *MPL* mutations commonly seen in classical MPN. Instead, a substantial proportion of CNL cases harbour mutations in genes such as *CSF3R* or *SETBP1* [1].
**Chronic Eosinophilic Leukaemia (CEL):** CEL is a rare MPN characterized by persistent eosinophilia in the peripheral blood. Furthermore, there must be evidence of clonality as well as abnormal bone marrow morphology. WHO criteria for other myeloid or lymphoid neoplasms, including MPN, MDS/MPN, MDS, and Myeloid/Lymphoid Neoplasms with eosinophilia and defining gene rearrangement (MLN-TK), are not met. For the exclusion of a variety of other myeloid neoplasms, like MLN-TK, cytogenetic and molecular studies should be used. Patients may experience symptoms related to organ involvement due to the accumulation of eosinophils in various tissues [1].
**Juvenile Myelomonocytic Leukaemia (JMML):** JMML is a unique form of paediatric MPN that primarily affects children. It is characterized by excessive production of granulocytes and monocytes and is associated with mutations in genes such as *NF1*, *NRAS*, *KRAS*, or *PTPN11* [1]. JMML is a highly aggressive disease and often requires haematopoietic stem cell transplantation as the only curative treatment.
**Myeloproliferative Neoplasm, Not Otherwise Specified (MPN-NOS):** MPN-NOS is a category that encompasses MPN that do not fit the diagnostic criteria of the aforementioned subtypes [1]. The genetic landscape of MPN-NOS is heterogeneous, and further research is needed to elucidate the molecular basis of these cases.

**Table 1:** Frequency* of recurrent somatic gene mutations in PV, ET and PMF [20].

	**Gene**	**Frequency (%)**		
**PV**		**ET**	**PMF**
Disease driver mutations	*JAK2* (V617F or exon12)	98	55	60
	*CALR*	0	25–30	20–30
	*MPL*	0	5–7	7–10
Clonal driver mutations	*TET2*	10–20	3–10	10–20
	*DNMT3A*	5–10	1–5	8–12
				
Mutations associated with progression	*ASXL1*	2–7	5–10	15–35
	*EZH2*	1–2	1–2	7–10
	*U2AF1*	<2	<2	7–10
	*SRSF2*	<2	<2	6–14
	*SF3B1*	2–3	2–5	5–7
	*IDH1/2*	1–2	1–2	5–6
	*TP53*	<2	<2	4–5
	*NRAS*	<2	<2	2–4
	*KRAS*	<2	<2	2
	*RUNX1*	<2	<2	2–3
				
Other	*CBL*	<2	<2	4
	*NFE2*	3–6	1–7	3–5
	*SH2B3*	2–9	1–3	2–5

Only gene mutations occurring with a frequency of ≥2 % in at least one entity are listed.

Mastocytosis and the above-mentioned MLN-TK represent separate disease families within WHO-HAEM5. MLN-TK comprise a family of diseases generated by fused and thereby dysregulated TK genes (rearrangements of *PDGFRA*, *PDGFRB*, *FGFR1*, *JAK2*, *FLT3*, *ETV6*::*ABL1* fusion, and other TK gene fusions). Eosinophilia is common, though not always present, in these conditions.

Mastocytosis includes cutaneous and systemic mastocytosis as well as mast cell sarcoma. Systemic mastocytosis is characterized by multifocal dense infiltrates of mast cells detected in sections of bone marrow and/or other extracutaneous organ(s). Minor criteria for systemic mastocytosis include the presence of atypical mast cells in bone marrow or other organs, activating *KIT* mutations, aberrant expression of specific antigens by mast cells, and a baseline serum tryptase concentration exceeding 20 ng/mL in the absence of a myeloid associated haematopoietic neoplasm [1].

## MPN vs. MDS/MPN: what’s the difference?

Distinguishing between classical MPN and MDS/MPN entities is a diagnostic challenge, particularly when considering the presence or absence of cytosis (increased cell counts), cytopenias and dysplasia (abnormal cell morphology). In classical MPN, the hallmark is cytosis characterized by elevated levels of mature myeloid cells in the peripheral blood. These disorders are often driven by mutations leading to the proliferation of specific blood cell lineages. Furthermore, dysplasias and atypias in the megakaryocytic lineage may also play a role in primary and post-PV as well as post-ET myelofibrosis and the MDS subtype MDS with increased blasts and fibrosis. In contrast, dysplasia refers to the presence of abnormal cell morphology, a hallmark feature of MDS. Dysplasia can manifest in various haematopoietic lineages, including erythroid, myeloid, and megakaryocytic cells. Interestingly, but also challenging in diagnostics: MDS/MPN share features of both groups [1].

## Myelodysplastic/myeloproliferative neoplasm (MDS/MPN)

MDS/MPN is a heterogeneous group of haematopoietic disorders defined by overlapping pathological and molecular features of both MDS and MPN. Clinically, MDS/MPN entities manifest with a variety of combinations of cytopenias and cytoses [1].

### Chronic myelomonocytic leukaemia (CMML)

CMML is the most common subtype of MDS/MPN and is characterized by sustained peripheral blood monocytosis. It represents a complex and biologically diverse entity with various combinations of somatic mutations involving genes related to epigenetic regulation, spliceosome function, and signal transduction pathways. The diagnosis of CMML is defined by specific criteria established by WHO-HAEM5 [1]:

Prerequisite criteria:

Persistent absolute (≥0.5 × 10^9^/L) and relative (≥10 %) peripheral blood monocytosis
Blasts constitute <20 % of the cells in the peripheral blood and bone marrow
Not meeting diagnostic criteria of CML or other MPN
Not meeting diagnostic criteria of myeloid/lymphoid neoplasms with eosinophilia and defining gene rearrangements (e. g. *PDGFRA*, *PDGFRB*, *FGFR1*, or *JAK2*)

Supporting criteria:

1. Dysplasia involving ≥ 1 myeloid lineages
2. Acquired clonal cytogenetic or molecular abnormality
3. Abnormal partitioning of peripheral blood monocyte subsets

For diagnosis, prerequisite criteria must be present in all cases. If monocytosis is ≥1 × 10^9^/L, one or more supporting criteria must be met. If monocytosis is <1 × 10^9^/L, supporting criteria 1 and 2 must be met [1].

### Diagnostics in CMML relies on monocytosis and molecular genetics

#### CMML: cytomorphology

The diagnosis of CMML is based on the detection of persistent absolute and relative monocytosis in the peripheral blood. Other conditions that could cause monocytosis must be excluded. Depending on the total leukocyte count, two variants are distinguished: MD-CMML (monocytosis dominant, white blood cell (WBC) count <13 × 10^9^/L) and MP-CMML (neutrophilic or eosinophilic proliferation dominant, WBC count ≥13 × 10^9^/L). Furthermore, CMML is categorized into CMML-1 (<5 % blasts in the blood or <10 % in bone marrow) or CMML-2 (5–19 % blasts in the blood or 10–19 % in bone marrow) [1].

#### CMML: immunophenotyping

Peripheral blood monocytes can be differentiated into classical monocytes (CD14^+^/CD16^-^), intermediate monocytes (CD14^+^/CD16^+^) and nonclassical monocytes (CD14^low^/CD16^-^). CMML patients show a characteristic increase (>94 %) in the classical monocyte subset. Therefore, immunophenotyping can be a valuable tool in distinguishing CMML from MDS and other MDS/MPN, aiding in the diagnostic process (supporting criterion 3) [1, 21].

#### CMML: cytogenetics

Clonal cytogenetic abnormalities are detected in approximately 30 % of CMML cases, although none are specific to the disease. Common cytogenetic abnormalities in CMML include trisomy 8, chromosome 7 alterations (such as –7 or del(7q)), loss of chromosome Y, and trisomy 21. Complex karyotypes are less common but may be associated with disease progression [1].

#### CMML: molecular genetics

Over 90 % of CMML patients harbour at least one molecular mutation, some of which have prognostic relevance. The genomic landscape of CMML includes mutations affecting DNA methylation (*TET2*, *DNMT3A*), RNA splicing (*SRSF2*, *SF3B1*, *U2AF1*, *ZRSR2*), signal transduction (*NRAS*, *KRAS*, *CBL*, *PTPN11*, *JAK2*), as well as transcription factors and nucleosome assembly (*SETBP1*, *RUNX1*) [1].

Prognosis in CMML can be assessed using the CMML-specific Prognostic Scoring System (CPSS) and CPSS-molecular, which incorporate clinical and molecular factors to stratify patients into risk groups, aiding in treatment decisions and prognostic counselling [22].

#### MDS/MPN-N

Myelodysplastic/myeloproliferative neoplasm with neutrophilia (MDS/MPN-N) represents a subtype that has replaced the term “atypical Chronic Myeloid Leukaemia (aCML).” This change underscores its MDS/MPN nature and avoids confusion with CML. MDS/MPN-N is characterized by sustained peripheral blood neutrophilia and neutrophilic left-shift. Specific essential diagnostic criteria defined by WHO-HAEM5 include [1]:

Peripheral blood leukocytosis ≥13 × 10^9^/ L, with neutrophilia and ≥10 % circulating immature myeloid cells (promyelocytes, myelocytes and metamyelocytes), as well as neutrophilic dysplasia
Hypercellular bone marrow with granulocytic predominance and granulocytic dysplasia, with or without dysplasia in the megakaryocytic and erythroid lineages.
<20 % blasts in blood and bone marrow
Not meeting diagnostic criteria for myeloproliferative neoplasms (specifically, exclusion of *BCR*::*ABL1* fusion), myeloid neoplasms with eosinophilia and defining gene rearrangement, CMML, or myelodysplastic/myeloproliferative neoplasm with *SF3B1* mutation and thrombocytosis

### Diagnostics of MDS/MPN-N

#### MDS/MPN-N: cytomorphology

Cytomorphology is a key diagnostic element to differentiate MDS/MPN-N from other MPN, CMML, as well as MDS, based on the criteria listed above.

#### MDS/MPN-N: cytogenetics

Karyotypic abnormalities are detected in 30–40 % of cases, with chromosomes 8 and 20 being the most commonly involved. Chromosomal abnormalities in MDS/MPN-M can sometimes be associated with disease progression [1].

#### MDS/MPN-N: molecular genetics

The diagnosis of MDS/MPN-N requires exclusion of the *BCR*::*ABL1* fusion gene, which may require careful evaluation to exclude cryptic rearrangements and/or alternate *BCR*::*ABL1* transcripts. MDS/MPN-N is characterized by mutations in genes such as *ETNK1* and *SETBP1*. Other mutations often involve *ASXL1*, *TET2*, and *DNMT3A*. The mutational profile of MDS/MPN-N shares similarities with other myeloid neoplasms, such as CNL, CMML, and MDS/MPN-NOS, emphasizing the importance of morphological criteria in distinguishing these entities [1].

### MDS/MPN-*SF3B1*-T (MDS/MPN with* SF3B1* mutation and thrombocytosis)

MDS/MPN-*SF3B1*-T, which is characterized by mutations in the *SF3B1* gene, represents a separate subset within the MDS/MPN spectrum. [1]. Based on its molecular characteristics, MDS/MPN-*SF3B1*-T demonstrates an overlap between MDS and MPN: on the one hand the characteristic *SF3B1* mutation in MDS/MPN-*SF3B1*-T is also defining the specific MDS subtype MDS-*SF3B1*. On the other hand, MDS/MPN-*SF3B1*-T reveals concomitant hallmark mutations of MPN such as *JAK2* mutations [1].

### MDS/MPN-NOS

MDS/MPN-NOS represents a category that encompasses cases not classifiable into specific subtypes and is diagnosed by exclusion and is characterized by a combination of cytopenia(s) and proliferative feature(s) in the peripheral blood, a combination of cell dysplasia and proliferative features in the bone marrow and a combination of mutations seen in proliferative and dysplastic myeloid malignancies.

In conclusion, MDS/MPN represents a complex group of haematological disorders with overlapping features of MDS and MPN. Detailed diagnostic criteria, genetic insights, and risk stratification systems are essential for accurate classification and the development of tailored treatment approaches for patients within this category. Ongoing research continues to expand our knowledge of these disorders, shedding light on their molecular intricacies and therapeutic potential.

## Conclusion

**Figure 2:**
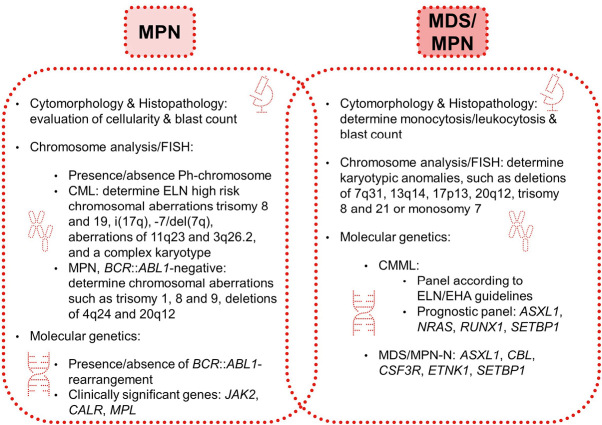
Diagnostic methods for MPN and MDS/MPN

Due to the overlapping characteristics between MDS/MPN and MPN, a careful and in-depth integrated diagnostic workflow is essential (see Fig. 2). Not only morphological features have to be investigated, but also clinical characteristics, morphology of bone marrow, cytogenetics, and molecular genetics.

In the future, there may be developments of artificial intelligence (AI) that increase quality and precision of the diagnostic workup. The automated analysis of bone marrow, guided by AI and an AI-guided diagnosis through genetic and molecular analyses, for example have high potential to support clinicians in the future [23, 24].

## References

[1] WHO Classification of Tumours Editorial Board. Haematolymphoid tumours [Internet; beta version ahead of print]. WHO classification of tumours series 2022; 5th ed.; vol. 11.

[2] Hehlmann R (2007). Chronic myeloid leukaemia. Lancet. 370(9584).

[3] Hochhaus A (2020). European LeukemiaNet 2020 recommendations for treating chronic myeloid leukemia. Leukemia. 34(4).

[4] Bower H (2016). Life Expectancy of Patients With Chronic Myeloid Leukemia Approaches the Life Expectancy of the General Population. J Clin Oncol. 34(24).

[5] White H E (2022). Standardization of molecular monitoring of CML: results and recommendations from the European treatment and outcome study. Leukemia. 36(7).

[6] Tefferi A (2023). Primary myelofibrosis: 2023 update on diagnosis, risk-stratification, and management. Am J Hematol. 98(5).

[7] Bacher U (2005). Conventional cytogenetics of myeloproliferative diseases other than CML contribute valid information. Ann Hematol. 84(4).

[8] Gangat N (2008). Cytogenetic studies at diagnosis in polycythemia vera: clinical and JAK2V617F allele burden correlates. Eur J Haematol. 80(3).

[9] Haferlach T (2008). The diagnosis of BCR/ABL-negative chronic myeloproliferative diseases (CMPD): a comprehensive approach based on morphology, cytogenetics, and molecular markers. Ann Hematol. 87(1).

[10] Sever M (2009). Cytogenetic abnormalities in essential thrombocythemia at presentation and transformation. Int J Hematol. 90(4).

[11] Tefferi A (2018). Revised cytogenetic risk stratification in primary myelofibrosis: analysis based on 1002 informative patients. Leukemia. 32(5).

[12] Cervantes F (2009). New prognostic scoring system for primary myelofibrosis based on a study of the International Working Group for Myelofibrosis Research and Treatment. Blood. 113(13).

[13] Gangat N (2011). DIPSS plus: a refined Dynamic International Prognostic Scoring System for primary myelofibrosis that incorporates prognostic information from karyotype, platelet count, and transfusion status. J Clin Oncol. 29(4).

[14] Guglielmelli P (2018). MIPSS70: Mutation-Enhanced International Prognostic Score System for Transplantation-Age Patients With Primary Myelofibrosis. J Clin Oncol. 36(4).

[15] Passamonti F (2010). A dynamic prognostic model to predict survival in primary myelofibrosis: a study by the IWG-MRT (International Working Group for Myeloproliferative Neoplasms Research and Treatment). Blood. 115(9).

[16] Tefferi A (2014). Integration of Mutations and Karyotype Towards a Genetics-Based Prognostic Scoring System (GPSS) for Primary Myelofibrosis. Blood. 124(21).

[17] Tefferi A (2018). MIPSS70+ Version 2.0: Mutation and Karyotype-Enhanced International Prognostic Scoring System for Primary Myelofibrosis. J Clin Oncol. 36(17).

[18] Tefferi A (2018). GIPSS: genetically inspired prognostic scoring system for primary myelofibrosis. Leukemia. 32(7).

[19] Vannucchi A M (2014). Mutation-Enhanced International Prognostic Scoring System (MIPSS) for Primary Myelofibrosis: An AGIMM & IWG-MRT Project. Blood. 124(21).

[20] Luque Paz D, Kralovics R, Skoda RC (2023). Genetic basis and molecular profiling in myeloproliferative neoplasms. Blood. 141(16).

[21] Selimoglu-Buet D (2015). Characteristic repartition of monocyte subsets as a diagnostic signature of chronic myelomonocytic leukemia. Blood. 125(23).

[22] Patnaik MM, Tefferi A (2022). Chronic myelomonocytic leukemia: 2022 update on diagnosis, risk stratification, and management. Am J Hematol. 97(3).

[23] Ryou H (2023). Continuous Indexing of Fibrosis (CIF): improving the assessment and classification of MPN patients. Leukemia. 37(2).

[24] Walter W (2022). Artificial intelligence in hematological diagnostics: Game changer or gadget?. Blood Rev.

